# Preliminary analyses of tryptophan, kynurenine, and the kynurenine: Tryptophan ratio in plasma, as potential biomarkers for systemic chlamydial infections in koalas

**DOI:** 10.1371/journal.pone.0314945

**Published:** 2024-12-19

**Authors:** Chien-Jung Chen, Benjamin Kimble, Astrid Van Aggelen, Shalini Fischer, Cheyne Flanagan, Amber Gillett, Jackie Reed, Jodie Wakeman, Merran Govendir

**Affiliations:** 1 Sydney School of Veterinary Science, The University of Sydney, Sydney, New South Wales, Australia; 2 Port Macquarie Koala Hospital, Koala Conservation Australia, Port Macquarie, New South Wales, Australia; 3 Australia Zoo Wildlife Hospital, Wildlife Warriors, Beerwah, Queensland, Australia; 4 Northern Rivers Koala Hospital, Friends of the Koala, East Lismore, New South Wales, Australia; Georgia State University, UNITED STATES OF AMERICA

## Abstract

Chlamydiosis is the major infectious disease responsible for significant morbidity and mortality in free-living koalas. Recently, it was reported that 28.5% of koalas infected with chlamydiosis were presented with no overt clinical signs. Identification and quantification of changes in plasma biomarkers’ fluctuations have the potential to enhance *C*. *pecorum* detection and facilitate the monitoring of therapeutic efficacy of antibiotics to treat this disease in koalas. Therefore, concentrations of the essential amino acid tryptophan, tryptophan’s metabolite kynurenine, and the kynurenine:tryptophan ratio were quantified by high-performance liquid chromatography in the plasma of clinically normal koalas (n = 35), koalas identified with chlamydial disease (n = 35) and koalas that had other non-chlamydial co-morbidities (n = 10). Results showed that there was a significant difference between the clinically normal versus diseased, and clinically normal versus ‘other’ (both p < 0.001) in kynurenine plasma concentrations and kynurenine:tryptophan ratio; and also between the clinically normal and diseased in tryptophan plasma concentrations (p = 0.001). Proposed reference ranges of tryptophan, kynurenine, and kynurenine:tryptophan ratio in koalas are: 4.27–10.4 μg/mL, 0.34–1.23 μg/mL, and 0.05–0.22, respectively. Proposed optimal cut-off points to differentiate between clinically normal and diseased are: ≤ 4.75 μg/mL (tryptophan), ≥ 0.88 μg/mL (kynurenine), and ≥ 0.12 (kynurenine:tryptophan); and ≤ 7.67 μg/mL (tryptophan), ≥ 1.18 μg/mL (kynurenine), and ≥ 0.16 (kynurenine:tryptophan) to differentiate between released/recovered and euthanised of the diseased/‘other’ koalas. Significant differences in haematological and biochemical analytes were in the plasma globulins between the clinically normal and diseased koalas (p = 0.01), and in alkaline phosphatase between the clinically normal and ‘other’ koalas (p = 0.03). Although these potential biomarkers, especially tryptophan, may not be specific for detecting *C*. *pecorum* from the rest of the population, kynurenine and the kynurenine:tryptophan ratio may have a role in identifying unhealthy koalas from the clinically normal ones, irrespective of the underlying cause.

## Introduction

Tryptophan (TRP), an essential amino acid, is active in the regulation of immune responses in humans and animals [1]. In mammals, TRP depletion can be due to one of four pathways with the major pathway being the metabolism of TRP to kynurenine (KYN), commonly referred as the TRP-KYN pathway, accounting for 95.0% of dietary TRP metabolism (90.0% in the liver and 5.00% outside the liver) [2]. The remaining 5.00% of TRP is metabolised as the serotonin synthesis pathway in the periphery and brain, in addition to other minor physiological pathways [3, 4]. The primary pathway of TRP depletion is activated as a response to stressors such as infection and inflammation [5, 6], oxidative stress [7, 8], and environmental toxicants [9, 10]. Therefore, the concentration of KYN (one of tryptophan’s metabolites) is expected to increase with depletion in the TRP concentration in times of stress and/or inflammation. In the clinically normal state, the TRP-KYN pathway is triggered by up-regulation of the enzyme tryptophan 2,3-dioxygenase (TDO2) in the liver, that converts TRP into formylkynurenine at a controlled rate [11, 12]. A diagram of the TRP-KYN metabolic pathway is found at https://journals.sagepub.com/doi/10.4137/IJTR.S12626 [13].

The pathophysiological significance of the KYN:TRP is that degradation of TRP and elevated KYN concentrations in the blood are associated with inflamed physiological states [3]. Inflammation induces the enzyme indoleamine 2,3-dioxygenase (IDO-1/IDO-2) by the inflammatory cytokine interferon gamma (IFN-γ), resulting in TRP biotransformation to active metabolites [3] such as KYN and melatonin (via the serotonin pathway). All three metabolites modulate immunity and induce anti-inflammatory responses [1]. Accumulation of KYN leads to cell cycle arrest of the effector T cells [14] and induces the differentiation of proinflammatory T helper type 17 cells [15]. Consequently, a significant increase in KYN metabolites, such as 3-hydroxykynurenine and quinolinic acid, are associated with neuro- [16, 17] and immune- [18–20] associated diseases, in humans and in animal models.

Chlamydiosis is the most prevalent infectious disease in koalas (*Phascolarctos cinereus*) [21]. This disease has devastating impacts on both individual koalas and the survival of whole free-living populations in South-East Queensland (QLD) and New South Wales (NSW) in Australia [21]. Depending on the site/s of infection, chlamydiosis can cause conjunctivitis and/or cystitis resulting in blindness, renal disease, infertility, and ultimately death [22, 23]. However, our recent retrospective study [24] reported that 28.5% of the infected koalas that are polymerase chain reaction (PCR) positive for chlamydial pathogens do not exhibit overt clinical signs, so establishing a reliable biomarker for detection of sub-clinical chlamydial infection, with *Chlamydia pecorum* (*C*. *pecorum*) being the predominant species, may allow for improved detection of disease onset and therapeutic monitoring of antibiotic treatment outcomes.

One of the key defense mechanisms against chlamydiosis is the depletion of endogenous TRP concentration to inhibit chlamydial development, known as TRP starvation [25]. Although chlamydial pathogens are known to uptake TRP as their main source of energy to survive, there are chlamydial species that have adapted to TRP starvation by transforming into nonreplicating but viable, persistent forms [26]. However, studies report that the interaction between TRP and the *C*. *pecorum* strain is unique compared to other *Chlamydia spp*., and to survive within a TRP-free environment, *C*. *pecorum* is equipped with TrpR (TRP operon repressor) which upregulates TRP biosynthesis when the TRP concentration is low [25, 27, 28]. Adequate amounts of TRP can be synthesised from KYN and anthranilic acid, and that this newly formed TRP binds to its cognate operator to stop transcription of the TRP operon when the desired concentration is reached [25].

Clinically healthy animals have innate amounts of TRP and KYN already in their blood, which are obtained from their diets [29]. Interspecies variation is evident in the intrinsic total TRP and KYN plasma concentrations, such as a TRP concentration of 12.9 ± 0.41 μg/mL (63.0 ± 2.0 μM) and KYN concentration of 0.45 ± 0.02 μg/mL (2.15 ± 0.12 μM) in clinically normal humans [30]; and clinically normal mice exhibit mean TRP concentrations of 26.9 μg/mL (131.5 μM) [31] and mean KYN concentrations of 0.35 μg/mL (1.68 μM) [32]. Studies have extensively investigated the KYN:TRP ratio with human neurological diseases [33] and lung cancer [34, 35]; more recently, a significant elevation in the KYN:TRP ratio has been associated with long COVID in humans [36–38].

To date, there has been no report on relationships between the KYN:TRP ratio and disease progression in animals. Studies thus far have explored variations in KYN:TRP ratio in response to stress in fish [39], chickens [40], and mice [41]; as well as rats with renal insufficiency [42–44], and cows with ketosis [45] and mastitis [46]. As chlamydial infections induce chronic inflammation in koalas [47], examining the KYN:TRP ratio may provide insights into the detection of subclinical disease and ways to enhance antibiotic treatments of *C*. *pecorum*. Since plasma TRP and KYN concentrations have not been quantified in clinically normal and diseased koalas, the aims of this study were to develop and validate an assay to quantify TRP and KYN concentrations in plasma of clinically normal and diseased koalas; to determine the reference ranges of TRP, KYN and the KYN:TRP ratio with clinically normal koalas; and to investigate the relationships in KYN, TRP, and the KYN:TRP ratio between clinically normal, diseased, and koalas with other co-morbidities associated with inflammation.

## Materials and methods

### Animals and housing

The study was approved by The University of Sydney Animal Ethics Committee protocol 2023/2259.

**Fig 1:**
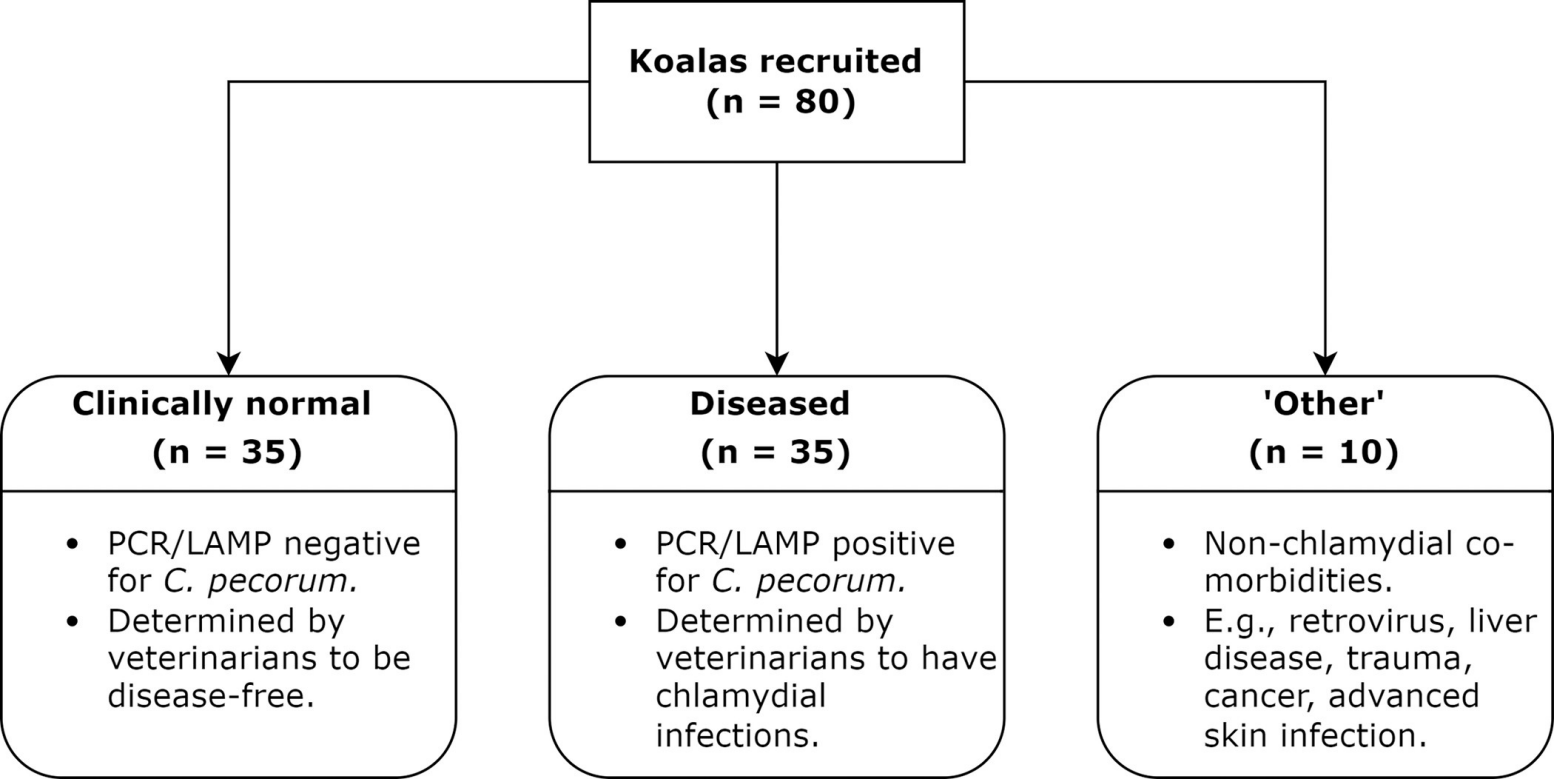
Summary of the koala groups recruited for this study.

A total of 80 koalas were recruited opportunistically for this study with the animals housed individually according to normal husbandry procedures used at the Australia Zoo Wildlife Hospital (Beerwah, QLD, Australia), the Port Macquarie Koala Hospital (Port Macquarie, NSW, Australia), and the Friends of the Koala Hospital (East Lismore, NSW, Australia). The recruited koalas were provided with fresh food *ad-lib* (various *Eucalyptus* spp. leaves) and water, perching material, shelter from inclement weather and daily removal of faeces and old browse. Of the 80 animals, 35 diseased koalas were admitted to the hospitals for chlamydiosis during the study period, 10 koalas were hospitalised for other co-morbidities, and 35 clinically normal koalas had samples taken opportunistically for other studies (Fig 1).

The health status of the koalas, either clinically normal, diseased with *C*. *pecorum*, or with other co-morbidities, were determined by veterinarians upon physical examination, and swabs for suspected cases of chlamydial infections were taken and confirmed by either PCR or loop-mediated isothermal amplification (LAMP) assay. For some koalas, blood was available for haematology and biochemistry. Koalas that were PCR/LAMP positive for *C*. *pecorum*, with clinical signs present, were categorised as ‘diseased’, and animals with other non-chlamydial co-morbidities, such as retrovirus, liver disease, joint infections, trauma, advanced alopecia and skin infection, leukaemia, and abdominal lymphoma were categorised as ‘other’.

### Sample collection, handling and blood component quantification

All koalas initially were examined under general anaesthesia. Sedation was achieved using up to 3 mg/kg alfaxalone (Alfaxan^®^-CD, RTU; Jurox Pty Ltd, Rutherford, NSW, Australia) as an intramuscular injection, while general anaesthesia was maintained with 1–2% isoflurane in 100% oxygen delivered via face mask or cuffed 4–4.5 mm endotracheal tube. The duration of anaesthesia was between 15 and 30 minutes to perform abdominal ultrasonography and swabbing of both conjunctivae and the urogenital tract for all animals, with or without clinical signs. A definitive diagnosis of chlamydiosis was confirmed by a positive PCR or LAMP result.

Whilst under general anaesthesia, 5 mL of blood was collected from each koala via venipuncture from the cephalic vein with a 23 g needle, of which 2 mL was collected into potassium EDTA tube for routine haematological analysis, and the remaining 3 mL was collected in a lithium heparin tube and centrifuged into plasma for biochemical analyses, and TRP and KYN quantification.

After the single blood collection from each koala for this study, the koalas classified as ‘diseased’ and ‘other’ were treated by the respective veterinarians (authors A.V.A., S.F., A.G., J.R., J.W.), who administered antibiotics, anti-inflammatories, and/or analgesics as required. No animals were sacrificed for this study; however, some koalas were euthanised due to poor treatment responses. The decision to euthanise was made by the veterinarians attending their respective patients, independent to this study.

Quantification of blood haematological values and biochemical analyte concentrations were conducted by Veterinary Pathology Diagnostic Services at the Sydney School of Veterinary Science, and Vetnostics at Macquarie Park in Sydney, to determine whether hydration status and the selected haematological or biochemical analytes significantly affected KYN and TRP plasma concentrations. The haematological values of interest were the total white blood cell count (WBC), red blood cell count (RBC), haematocrit (HCT), neutrophil count (Neut), lymphocyte count (Lymph), and monocyte count (Mono). The biochemical analytes investigated were alkaline phosphatase (ALP), alanine transaminase (ALT), albumin (ALB), creatinine (CRE), globulins (Glob), total protein (TP), gamma-glutamyl transferase (GGT), and blood urea nitrogen (BUN).

Most freshly collected samples, and only a few frozen, were transported on dry ice from all the koala hospitals to The University of Sydney, where the samples were thawed, and then assayed for TRP and KYN quantification within 72 h of arrival. Other freshly collected samples were either delivered at 4˚C for a maximum of 48 h, then assayed, or stored at -20˚C before thawing at ambient room temperature (∼20°C) for analysis.

### Tryptophan and kynurenine analysis in koala plasma

Tryptophan (catalogue number T0254) and KYN (catalogue number K8625), acetic acid (catalogue number 695092), trichloroacetic acid (TCA; catalogue number T6399), and theophylline (catalogue number T1633), as the internal standard (IS), were purchased from Sigma-Aldrich (Castle Hill, NSW, Australia). Ammonium acetate (catalogue number A16343.30) and methanol (MeOH; catalogue number A452) were purchased from Thermo Fisher Scientific (Macquarie Park, NSW, Australia). Purified water was retrieved from the Milli-Q water purification system (Merck Millipore, Burlington, MA, USA).

A reversed-phase high-performance liquid chromatography (HPLC) system from Shimadzu (Rydalmere, NSW, Australia) that included a LC-20AT system controller, a DGU-20A_3_ degassing unit, a SIL-20A HT auto sampler, a RF-10A XL fluorescence detector, a SPD-20A UV detector, and a CTO-20A column oven was used.

The separation method was modified from a previous method [48], and then optimised to suit the complexity of the koala plasma. Chromatographic separation was performed by Agilent Polaris C_18_-A, 5 μm, 150 mm × 4.6 mm column that was maintained at ambient room temperature (∼20˚C). The gradient mobile phases composed of A = 4.76% MeOH in 20mM ammonium acetate (pH 4.5) in water, and B = 80% MeOH in 20mM ammonium acetate (pH 4.5) in water with a flow rate of 1.2 mL/min. The initial mobile phase composition was maintained at 5% solvent B for 1 min (0–1 min), increased linearly from 5–50% solvent B in 6 mins (1–7 min), held for 1 min (7–8 min), then returned to the initial composition at 5% for 5 mins (8–13 min) for the chromatograph column equilibrium. The fluorescence detector was set at an excitation wavelength of 297 nm and an emission wavelength of 347 nm to detect TRP, with the UV detector at 360 nm to detect the KYN and the IS. The total run time for each sample was 13 min.

### Sample preparation

Since endogenous TRP and KYN concentrations were detected in blank koala plasma, fresh calibration standards were prepared daily in phosphate buffered saline (PBS) rather than using blank koala plasma. Samples for the calibration curve were spiked with the TRP and KYN working solutions (500 μg/mL in water) from the stock solutions (2 mg/mL in MeOH) to achieve concentrations of 0.63, 1.25, 2.50, 5.00, 10.0, and 20.0 μg/mL. A stock solution of the IS (2 mg/mL theophylline) was prepared fresh in MeOH and was further diluted fresh to give a working solution of 500 μg/mL before analysis. The same TRP, KYN and IS stock solutions were used for the quality control (QC) samples of low, medium, and high concentrations (2.50, 5.00 and 20.0 μg/mL) for assay validation in koala plasma. To calculate the area under the curve (AUC) of TRP and KYN in QC samples, the endogenous concentrations were subtracted using the following equations:

AUCofTRPforQC=(AUCofTRPinspikedQCsample)−(AUCofTRPinblankplasma)

AUCofKYNforQC=(AUCofKYNinspikedQCsample)−(AUCofKYNinblankplasma)

A protein precipitation extraction method was undertaken to separate the desired compounds from the matrix, by adding 50 μL of 10% TCA, 200 μL of MeOH with 7 μL of IS working solution into 100 μL of each PBS sample. The sample was vortexed then centrifuged at 20,854 × *g* for 10 min before injecting 10 μL of the supernatant into the HPLC system. This was repeated for a total of three times for each calibration and QC sample.

### Assay validation

The assay analytical conditions were defined for specificity, linearity, the lower limit of detection (LLOD), the lower limit of quantitation (LLOQ), and the analyte stability and recovery from the samples, with the assay validated for accuracy and precision [49]. The specificity was determined by identifying the peaks and retention times of the compounds of interest in plasma samples. The TRP, KYN, and the IS peaks were consistently observed with the retention times of ≈ 6.10 mins (fluorescence detection), ≈ 3.40 mins (UV detection), and ≈ 7.00 mins (UV detection), respectively.

Linearity was evaluated by analysing the prepared calibration solutions of TRP and KYN in PBS at concentrations of 0.63, 1.25, 2.50, 5.00, 10.0, and 20.0 μg/mL to generate a universal calibration curve (average of the three linear regressions) in the form of y = ax + b.

Sensitivity was based on the LLOD and the LLOQ values, which were calculated using the formulae [50]:

$$LLOD=(\frac{\sigma }{X})\times 3$$

$$LLOQ=(\frac{\sigma }{X})\times 10$$

where σ = the standard deviation of the *y*-axis intercepts and X = the average of the calibration curve gradients.

A weighting factor (1/x) was applied to ensure that the observations were not overfitted, especially at the lower concentrations.

The intra- and inter-day accuracy and precision was determined using the same TRP and KYN QC concentrations of 2.50, 5.00, and 20.0 μg/mL in triplicates across three days. Accuracy (%) was calculated using the formula [49]:

$$Accuracy\;(\%)=\frac{Estimated\;QC\;concentration}{Actual\;QC\;concentration}\times 100$$

Precision (CV %) was obtained by dividing the standard deviation by the mean estimated concentration, multiplied by 100 [49]. The accuracy and precision of the calculated values at each concentration was within ± 15% of the expected value [49].

The stability of TRP and KYN in the koala plasma was investigated under the following storage conditions in duplicates with the IS spiked at 10 μg/mL: ambient room temperature (∼20˚C) for 5 h; 4˚C for 2, 7, 9 and 14 days; and -20˚C for 3, 8, 15, 29 and 43 days. The mean ‘stable’ concentration obtained was within ± 15% of the initial concentration [49].

The recovery of TRP and KYN was calculated by pre-spiking the TRP and KYN concentrations to 2.50, 5.00, and 20.0 μg/mL, while the concentration of IS was 10 μg/mL in the blank plasma. Identical TRP, KYN and IS concentrations were vortexed into the Milli-Q water as a separate batch to calculate the average recovery percentage with the equation:

$$\%\;average\;recovery=100\times \frac{\left(Mean\;AUC\;of\;spiked\;plasma-Mean\;AUC\;of\;blank\;plasma\right)}{Mean\;AUC\;of\;spiked\;control\;in\;water}$$

All samples in the plasma and water followed the sample preparation and extraction methods described previously, which were then analysed as triplicates.

### Statistical analyses

All data analyses were performed in jamovi (version 1.6, Australia) at https://www.jamovi.org/ [51]. Descriptive statistics, such as counts and percentages, were conducted to explore the data distribution of the four variables, namely sex (male/female), maturity (juvenile/adult [≥ 2 years of age]), location (NSW/QLD), and koala health status (clinically normal/diseased/‘other’). One-way analysis of variance (ANOVA), coupled with Dwass-Steel-Critchlow-Fligner pairwise comparison, were conducted to determine the statistical significance between the hydration haematological values and biochemical analytes concentrations, and the clinically normal/diseased/‘other’ groups. Pearson’s correlation analysis was performed to investigate the association between KYN:TRP ratio and some of the haematological (WBC, Mono, Lymph) and biochemical (ALP, ALT, ALB, CRE, GGT, Glob) analytes. The Shapiro-Wilk test was used to investigate the normality of the data. The Welch’s *t*-test and the Mann-Whitney *U* (non-parametric) test were used to investigate significant differences between the variables and KYN, TRP, and KYN:TRP ratios. For all statistical analyses, the level of significance was set at p < 0.05.

### Reference ranges of clinically normal koalas

The 95% reference interval (reference range) was calculated with the equation conventionally used for data with a normal distribution as (mean ± 1.96 × SD), where 1.96 = z-score, and SD is the standard deviation [52, 53]. For non-parametric datasets, log transformations or Box-cox transformation were used to transform into parametric datasets before calculating the reference intervals [54]. Data transformations and reference interval calculations were performed on the MedCalc Statistical Software (version 22.013, Belgium) at https://www.medcalc.org/ [55].

### Sensitivity, specificity, and predictive values

The sensitivity, specificity, positive predictive value (PPV), and negative predictive value (NPV) of TRP and KYN concentrations, and the KYN:TRP ratio were calculated using conventional formulae corresponding to the cells in Table 1 [56]:

Sensitivity%=[a/(a+c)]×100

Specificity%=[d/(b+d)]×100

Positivepredictivevalue(PPV)=[a/(a+b)]×100

Negativepredictivevalue(NPV)=[d/(c+d)]×100

**Table 1:** Two-by-two table for calculating the sensitivity, specificity, NPV and PPV.

	Diseased (*C*. *pecorum*)	Clinically normal
Positive according to the proposed reference range	**a**	**b**
	(True positive)	(False positive)
Negative according to the proposed reference range	**c**	**d**
	(False negative)	(True negative)

### Receiver Operating Characteristic (ROC) analyses

The optimal cut-off points for KYN, TRP, and the KYN:TRP ratio were determined based on the Youden’s index [57], which favours the cut-off point with the best trade-off between sensitivity and specificity, using the Receiver Operating Characteristic (ROC) analysis [58]. All ROC curves and the optimal cut-off points were performed in jamovi.

## Results

### Assay validation

The accuracy and precision of the estimated KYN and TRP concentrations in intra-day and inter-day QC samples, spiked at 2.5, 5 and 20 μg/mL, are within ± 15% (S1 Table). The calculated LLOD and LLOQ values were 0.05 μg/mL and 0.15 μg/mL for KYN, and 0.03 μg/mL and 0.08 μg/mL for TRP, respectively.

The stabilities of KYN and TRP were evaluated at ambient room temperature (∼20˚C) over 5 h, 4˚C over 14 days, and -20˚C over 43 days, with both analytes determined to be unstable after 2 h at ambient temperature, 14 days at 4˚C, and 29 days at -20˚C.

The high recovery of KYN, obtained from concentrations of 2.50, 5.00 and 20.0 μg/mL, were 94.9 ± 8.85%, 107 ± 15.3%, and 99.9 ± 14.3%, respectively. Comparably, the recovery of TRP, with the same concentrations, yielded 83.8 ± 7.24%, 100 ± 4.38%, and 96.6 ± 5.83%, respectively.

### Koala demographics

A total of 80 koalas were recruited, comprising of 48.8% males and 51.3% females. Of these, 90.0% were adults and 10.0% were juveniles (< 2 years of age). Geographically, 80.0% were located in NSW and 20.0% in QLD. The proportion of clinically normal (43.8%) and diseased koalas with chlamydiosis (43.8%) were identical, while 12.5% of the koalas had ‘other’ co-morbidities (see S2 Table for demographic information, details on specific ‘other’ presentations, and the KYN and TRP concentrations).

### Observations in kynurenine and tryptophan concentrations

There was no significant difference in the potential biomarkers (KYN, TRP and KYN:TRP ratio) between clinically normal or diseased males and females.

A significant univariable difference was observed in the TRP concentrations between the clinically normal juvenile and adult koalas (juveniles had higher TRP concentrations than adults) when calculated using the Welch’s *t*-test (p = 0.04) as presented in S3 Table. However, there was no difference between the KYN, TRP, or KYN:TRP ratio and the maturity within the diseased or ‘other’ koalas.

There were no differences in KYN and TRP concentrations, and the KYN:TRP ratio, between NSW and QLD in the clinically normal group, nor in the diseased group.

**Fig 2:**
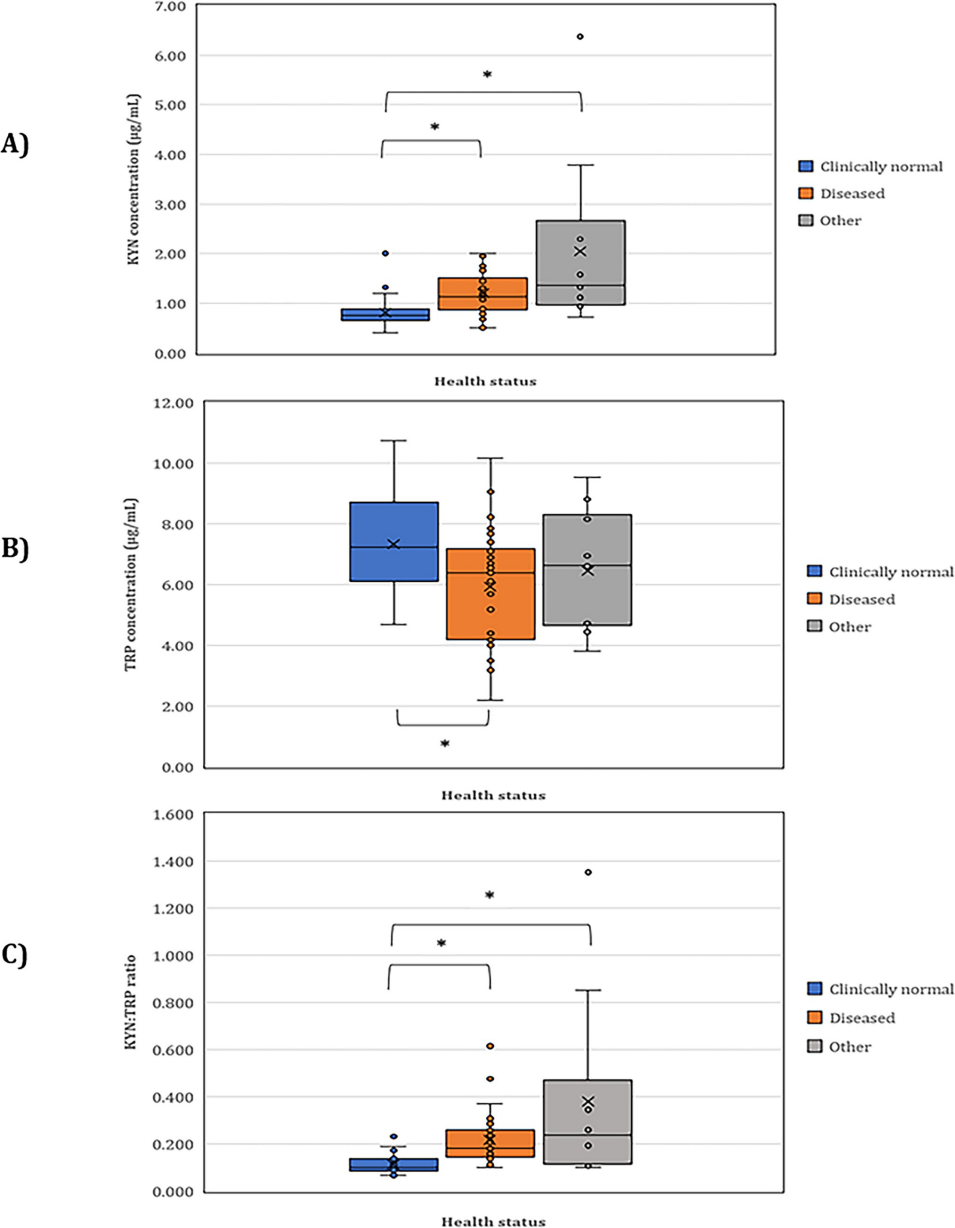
Box and whiskers plot of KYN, TRP, and KYN:TRP ratio in clinically normal (n = 35), diseased (n = 35) and ‘other’ koalas (n = 10). (A) KYN concentrations. (B) TRP concentrations. (C) KYN:TRP ratio. The outlier in the diseased group had both eyes and UGT clinical signs with mammary mass, and the outlier in the ‘other’ group had leukaemia. * = statistically significant.

A summary of the median and range of KYN, TRP, and the KYN:TRP ratios in clinically normal, diseased, and ‘other’ koalas are presented in Table 2. There is a significant difference between the KYN and KYN:TRP in clinically normal versus diseased, and clinically normal versus ‘other’ (all p < 0.001) but not between diseased versus ‘other’ in KYN and the KYN:TRP ratio (Fig 2). There is a significant difference between the clinically normal versus diseased in TRP (p = 0.001) only.

**Table 2:** Summary of the median and range of TRP, KYN, and the KYN:TRP ratios in clinically normal, diseased, and ‘other’ koalas.

Health status	Median (range) of KYN	Median (range) of TRP	Median (range) of KYN:TRP ratios
Clinically normal	0.76 (0.40–1.20) *	7.22 (4.68–10.76)	0.10 (0.07–0.23)
Diseased	1.13 (0.50–2.01)	6.38 (2.20–10.17)	0.18 (0.10–0.48) *
‘Other’	1.37 (0.71–2.29) *	6.63 (3.81–9.55)	0.24 (0.10–0.85) *

* = outlier/s excluded from calculation.

#### Haematological values and biochemical analytes

The haematological values and biochemical analytes concentrations (S4 Table) showed no significant differences in the haematological values and biochemical analytes except for Glob between the clinically normal and diseased koalas (p = 0.01), and in ALP between the clinically normal and ‘other’ koalas (p = 0.03) (S5 Table). There were no statistical differences in the haematological values and biochemical analytes concentrations associated with hydration status between the clinically normal, diseased, and ‘other’ koalas.

There was no correlation between KYN:TRP ratio and some other biochemical and haematological inflammatory biomarkers when Pearson’s correlation analysis was conducted with koalas in the diseased group, and separately with clinically normal koalas. When analysed with koalas in the diseased or ’other’ groups together, correlations were observed between KYN:TRP ratio and WBC (Pearson’s r value of 0.805, p < 0.001); KYN:TRP and Lymph (0.671, p < 0.001); and KYN:TRP and ALT (0.621, p = 0.001).

### Reference ranges of KYN, TRP, and the KYN:TRP ratio in clinically normal koalas

The proposed reference ranges of KYN, TRP, and the KYN:TRP ratio in clinically normal koalas are 0.34–1.23 μg/mL, 4.27–10.4 μg/mL, and 0.05–0.22, respectively. Concentrations of KYN, TRP, and the KYN:TRP ratios of all koalas, along with the hypothesised reference ranges, are presented in S1 Fig.

### Sensitivity, specificity and predictive values

The sensitivity, specificity, PPV and NPV of the proposed KYN, TRP, and KYN:TRP ratio reference ranges were calculated using the conventional formulae, with the reference ranges yielding high specificity and PPV across the three biomarkers (Table 3).

**Table 3:** Sensitivity, specificity, and predictive values of the proposed KYN, TRP, and KYN:TRP ratio references ranges for screening diseased koalas.

Biomarker (proposed reference range)	Sensitivity (%)	Specificity (%)	PPV (%)	NPV (%)
KYN (0.34–1.23 μg/mL)	40.0	94.3	87.5	61.1
TRP (4.27–10.4 μg/mL)	28.6	97.1	90.9	57.6
KYN:TRP ratio (0.05–0.22)	40.0	97.1	93.3	61.8

PPV = positive predictive value; NPV = negative predictive value.

### Receiver Operating Characteristic (ROC) analyses of KYN, TRP, and the KYN:TRP ratio

The optimal cut-off points of KYN, TRP and the KYN:TRP ratio are shown in Table 4. These values were derived from the clinically normal, diseased, and ‘other’ koalas with the combined ROC curves presented in S2 Fig. Further ROC analysis was performed to decide cut-off points of KYN, TRP and the KYN:TRP ratio for survival outcome (i.e., released versus euthanised) of the koalas in the diseased and ‘other’ groups in S3 Fig.

**Table 4:** Optimal cut-off points and its sensitivity, specificity, and predictive values of the proposed KYN, TRP, and KYN:TRP ratio in clinically normal versus diseased/‘other’, and released/recovered versus euthanised in diseased/‘other’ koalas.

Comparison Group	Optimal cut-off point	Sensitivity (%)	Specificity (%)	PPV (%)	NPV (%)	Youden’s index	AUC
**Clinically normal vs. diseased/ ‘other’**							
KYN (μg/mL)	≥ 0.88	77.8	74.3	79.6	72.2	0.52	0.81
TRP (μg/mL)	≤ 4.75	35.6	97.1	94.1	54.0	0.33	0.68
KYN:TRP ratio	≥ 0.12	88.9	71.4	80.0	83.3	0.60	0.87
**Released/recovered vs. euthanised**							
KYN (μg/mL)	≥ 1.18	89.5	68.4	73.9	86.7	0.58	0.73
TRP (μg/mL)	≤ 7.67	94.7	31.6	58.1	85.7	0.26	0.55
KYN:TRP ratio	≥ 0.16	100	42.1	63.3	100	0.42	0.73

PPV = positive predictive value; NPV = negative predictive value; AUC = area under the curve.

## Discussion

This is the first study to investigate TRP and KYN concentrations, and the KYN:TRP ratio in koala plasma between clinically normal and general disease states. This was a pilot study, and therefore reference ranges and the optimal cut-off points for KYN, TRP and the KYN:TRP ratio in the plasma are proposed, however, a larger sample size would increase the confidence of these ranges.

An accurate and precise assay to detect and quantify KYN and TRP concentrations was optimised for koala plasma. Other studies have developed reversed-phase HPLC assays for the same purpose in human plasma [59, 60] and serum [48, 61–64], however, modifications requiring UV detection of KYN, and fluorescence detection of both TRP and the IS, were necessary to overcome the interference of the endogenous peaks inherent in the plasma of clinically normal koalas [65, 66]. Additionally, developing an assay that could analyse both substrates simultaneously was critical due to their instability after two hours at ambient room temperature (∼20˚C).

Although TRP and KYN blood concentrations have been quantified in many species [30, 31, 46, 67–69], and the KYN:TRP in others [39, 41, 70], there are no studies proposing reference ranges for these potential biomarkers within the biological matrix of any animal species.

In comparison to the human plasma TRP reference range of 8.76–15.2 μg/mL (42.9–74.4 μmol/L) [71], the proposed koala plasma TRP reference range is lower at 4.27–10.4 μg/mL. The lower range in the koala could be due to their diet that is restricted to certain *Eucalyptus* spp. leaves known to be low in TRP content (1.10% and 3.60% in young and mature leaves, respectively) [72]; compared to human dietary sources of TRP such as the cow milk protein, α-lactalbumin, containing around 5.80% TRP [73].

The human plasma KYN reference range of 0.25–0.85 μg/mL (1.20–4.10μM) [74] is slightly lower than the proposed plasma KYN reference range at 0.34–1.23 μg/mL in koalas. As the main source of endogenous KYN concentration is synthesised in the liver predominantly by tryptophan 2,3-dioxygenase (TDO2) [75], perhaps higher KYN concentrations in koalas could be the result of greater liver activity due to their needs of metabolising plant secondary metabolites (PSMs) from the diet. Koalas have some very active hepatic metabolism pathways as a result of high cytochrome P450 monooxygenases (CYP) content in their livers [76], most noticeably CYP2C [77]. Another possibility for koalas having higher endogenous KYN concentrations than humans is due to their lower metabolic rate [78, 79], which may result in slower breakdown/excretion of KYN.

As a result of higher KYN and lower TRP plasma concentrations detected in clinically normal koalas when compared to humans, it is expected that the proposed KYN:TRP ratio reference range of 0.05–0.22 in clinically normal koalas is greater than in healthy humans of < 0.06 (calculated from the human TRP and KYN reference ranges above in μg/mL).

A common characteristic of all three potential biomarkers was high specificity (range of 94.3–97.1%) and low sensitivity (28.6–40.0%); in addition to high PPV (87.5–93.3%) and relatively lower NPV (57.6–61.8%) (Table 3). Altogether, high specificity and high PPV indicate that the proposed reference ranges have high probabilities of avoiding false positives and identifying true positives [80], when the koalas deemed positive are indeed diseased. Conversely, there is a relatively higher probability that the infected koalas could obtain KYN:TRP ratios within the proposed reference range (false negatives) with low sensitivity and low NPV. Practically, this implies that if a koala has a low KYN:TRP ratio, it cannot be discounted for being diseased and should be monitored, with the KYN:TRP ratio repeated later. However, if a high ratio is calculated, there is a high probability that inflammation is present.

For screening chlamydial infections, higher specificity can prevent unnecessary psychological stress and costly treatments [81]. This is reflected in previous studies for screening chlamydial infections in humans, where the reported specificity has been higher than the sensitivity [82–84]. In koalas, minimising the possibility of false positives is critical to prevent unnecessary stress, cost, and the need for invasive treatment procedures under general anaesthesia. However, many koalas with sub-clinical infections could be missed if relying solely on an elevated KYN:TRP ratio.

The ROC analyses were conducted on the TRP and KYN concentrations, and the KYN:TRP ratio in clinically normal versus diseased and ‘other’ koalas; and also on the potential biomarkers of released/recovered koalas versus euthanised of the diseased and ‘other’ koalas. For both, the optimal cut-off points demonstrated good sensitivity, specificity, and predictive values for both KYN and the KYN:TRP ratio but not for TRP (Table 4). As AUCs of 0.70 or above are acceptable, and values above 0.80 considered having excellent accuracy [85], only the KYN and KYN:TRP ratio optimal cut-off points should be considered. Therefore, koalas with KYN concentrations of ≥ 0.88 μg/mL and/or KYN:TRP ratios of ≥ 0.12 have high probabilities of being diseased or ‘other’. Further, koalas with KYN concentrations of ≥ 1.18 μg/mL and/or KYN:TRP ratios of ≥ 0.16 have greater chances of being euthanised due to disease than being released. While the ROC analysis is an attempt to determine the survivability of infected koalas, more comprehensive data, such as the severity of infections, should be collected and considered for greater confidence.

### Tryptophan plasma concentrations

Although plasma TRP concentrations were significantly higher in the clinically normal than the diseased koalas (Fig 2B and S6 Table), the concentration could be dependent on the stage of infection due to the nearly complete TrpR of *C*. *pecorum* pathogens, whereby TRP biosynthesis is upregulated by the pathogen when the concentration is low [25, 27, 28]. However, to date, TRP threshold and the change in TRP concentration upon the activation of the TrpR (whether it surpasses or maintains its threshold concentration) are undefined. Furthermore, there are 11 koalas, 10 in the diseased group and 1 from the ‘other’ group with TRP concentrations below the proposed reference range (< 4.24 μg/mL). Of these 11 koalas, the five were euthanised, five diagnosed with chlamydiosis were later released, and one had trauma and on recovery was released (S2 Table). As anticipated, koalas with the TRP concentrations below the proposed reference range were predominantly in the cohort afflicted by the disease. However, a low TRP concentration is not a convincing indication of disease severity, consequently, considering TRP alone as a potential biomarker for *C*. *pecorum* is not recommended.

### Kynurenine concentrations and the KYN:TRP ratio

Higher concentrations of KYN and the KYN:TRP ratio are associated with koalas in the diseased and ‘other’ groups in comparison to the clinically normal. Expectedly, a narrow distribution was observed in the KYN:TRP ratio of the clinically normal, followed by the diseased, then the ‘other’ group with the widest distribution (Table 2 and Fig 2C). The anomaly for KYN and the KYN:TRP ratio in the ‘other’ group was the same koala (K62) with leukaemia, while the other anomaly for the KYN:TRP ratio in the diseased group (K39) was positive for *C*. *pecorum*, from swabs collected from the conjunctiva and UGT, with a mammary mass. Although a significantly higher KYN:TRP ratio does not consistently indicate the presence of neoplasia, as demonstrated in K74 with abdominal lymphoma (KYN:TRP of 0.28). Conversely, there was no statistical significance in KYN, TRP, and the KYN:TRP ratio between diseased and the ‘other’ koalas, suggesting that the potential biomarkers may not be specific for *C*. *pecorum* infections; though they may indicate the severity of inflammation as both koalas diagnosed with neoplastic disease were later euthanised.

The TRP, KYN and KYN:TRP did not appear to be distorted due to hydration status, nor were there any obvious differences in hepatic and/or renal function between the clinically normal, diseased, and ‘other’ groups. While a significant increase in Glob in the diseased group (p = 0.01) and a reduction in ALP in the ‘other’ when compared to the clinically normal koalas (p = 0.03) were observed, and even though changes in these biomarkers could occur in dehydrated animals, elevated concentrations of CRE and BUN are more convincing dehydration markers [86, 87]. A possible factor for the fluctuation of Glob is likely due to the body’s response to inflammation in the diseased, as many globulins are well-established inflammatory markers [88, 89]. Whereas, the decline in ALP concentration has been attributed to dietary magnesium deficiency [90] as a consequence of loss of appetite from co-morbidities such as leukaemia, abdominal lymphoma, and trauma in the ‘other’ koalas. Although there was no significant difference in ALB concentration and the health status of the koalas, TRP is known to bind exclusively to ALB in the plasma; whereby 10.0–15.0% of the total plasma TRP existing in an unbound state in rats [91, 92], and only 6% in kangaroos [68]. While the unbound proportion of TRP is undefined in the koala, this study exclusively quantified the total plasma TRP concentrations and did not distinguish between TRP bound to plasma proteins and the unbound form.

Of the inflammatory biomarkers analysed, the Pearson’s correlation analysis determined a strong positive relationship between KYN:TRP ratio and WBC (0.805, p < 0.001), Lymph (0.671, p < 0.001), and ALT (0.621, p = 0.001) for the koalas in diseased or ‘other’ groups. This was expected as the activation of the TRP-KYN pathway, accompanied with an elevation in WBC, Lymph and ALT, are common responses to systemic and/or liver inflammation [93, 94]; though no correlations were evident for the koalas in the diseased group only, nor the clinically normal only, which indicated KYN:TRP to be a promising non-specific inflammatory biomarker in koalas.

One of the limitations of this study was the sample size; specifically in demographic variables, such as number of juvenile (n = 6), samples from a QLD wildlife hospital (n = 16), and ‘other’ with non-chlamydial diseases (n = 10). As koala samples are collected opportunistically, a larger sample size was restricted due to exhaustion of resources. As a result of limited access to koalas due to their endangered status, opportunistic sampling was used in this study. Consequently, while associations between KYN, TRP, and KYN:TRP and chlamydial infection were observed, the causal relationships between these biomarkers and the infection have yet to be established. Another limitation is that the haematology and biochemistry results were only available for 35 and 36 koalas, respectively, from a total of 80 koalas. This can also be resolved with additional increased time and resource allocations to ensure completeness of data. The final limitation was that some diseased koalas may have had elevated endogenous glucocorticoid levels. As a response to stressors, like other species, koalas will increase glucocorticoid production, such as cortisol, from their hypothalamic-pituitary-adrenal (HPA) axis [95]. Endogenous glucocorticoids have anti-inflammatory properties and can induce tryptophan 2,3-dioxygenase (TDO2), thereby increase TRP depletion and elevate the KYN concentration [96, 97]. Only one of the recruited koalas was administered exogenous prednisolone to treat UGT infections (K17), yet there was no significant decrease in TRP concentration or increase in KYN concentration when compared to the other diseased koalas. Even with the study design assisting to minimise the effect of glucocorticoids on KYN concentrations by collecting blood samples under general anaesthesia, the various amount of stress endured by the koalas may have impacted the results. Even though other anti-inflammatory drugs, such as non-steroidal anti-inflammatory drugs (NSAIDs), could also affect TRP and KYN blood concentrations [98], these drugs were not administered prior to blood collection from the recruited koalas.

Further investigation on these potential biomarkers, along with serotonin and quinolinic acid as these metabolites are also indicators of inflammation [99], in a larger sample size with the disease severity documented would draw more robust conclusions. This can also determine whether there is a change in the KYN:TRP during treatment and whether these TRP metabolites have the potential to be reliable prognostic markers for antibiotic treatment success for chlamydiosis in this species. Examination of the KYN:TRP may also be helpful to monitor response to treatment for other non-infectious, inflammatory conditions, such as an animal with arthritis response to non-steroidal anti-inflammatory agents. Correlation analysis could also be conducted between the KYN:TRP ratio and other inflammatory biomarkers, including the acute phase proteins such as C-reactive protein (CRP) and serum amyloid A protein [100], cancer antigen 125 (CA-125), and erythrocyte sedimentation rate (ESR) [101], to determine the strength of the associations. A novel strategy to improve antibiotic efficacy against chlamydial pathogen is oral supplementation of TRP, which may reactivate dormant chlamydial pathogens into their metabolically active states and preventing them to revert to their dormant state [102]. Antibiotics are efficacious against metabolically active pathogens; however, it is also recognised that they can also induce dormancy [103]. Therefore, exploring the treatment efficacy of administering TRP supplementation with antibiotics against dormant chlamydial pathogens could be a new avenue for research.

## Conclusion

This is the first study to successfully detect and quantify plasma KYN and TRP concentrations in koala plasma, and to investigate the associations between clinically normal, diseased, and koalas with other co-morbidities. The koala reference ranges of KYN, TRP, and the KYN:TRP ratio are also proposed, in addition to the optimal cut-off points to distinguish between clinically normal versus diseased and released versus euthanised of diseased koalas to suggest the chances of recovery. The study concluded that although the potential biomarkers may not be specific for detecting *C*. *pecorum* from the rest of the population, this study strengthened the level of confidence in the capability of KYN and the KYN:TRP ratio to identify unhealthy koalas from the clinically normal, regardless of the cause. Until further studies demonstrate high confidence in detecting *C*. *pecorum* pathogens with biomarkers, PCR still remains the golden standard to identify chlamydial presence in koalas.

## Supporting information

S1 Table Estimated plasma KYN and TRP concentrations of the QC samples as triplicates spiked with KYN and TRP at different concentrations (2.50, 5.00, 20.0 μg/mL), with the accuracy (%) and precision (%) of each intra-day, and the mean ± SD for the inter-day results across three days. (PDF)

S2 Table Demographic information and the KYN and TRP concentrations of the koalas recruited (n = 80). (PDF)

S3 Table Univariable difference of the KYN, TRP, and KYN:TRP ratio between clinically normal juveniles (n = 6) versus adults (n = 29). Non-normally distributed data was analysed using non-parametric test. ^1^ = reference category; ^α^ = non-normal distribution; ^β^ = normal distribution; SD = standard deviation. Statistically significant value is bolded.
(PDF)

S4 Table Haematological values and biochemical analytes concentrations in clinically normal (n = 11), diseased (n = 20), or ‘other’ (n = 5) koalas. Reference ranges: WBC = 2.6–9.8 × 10^9^/L; RBC = 2.9–4.4 × 10^12^/L; HCT = 0.31–0.45 L/L; Neut = 0.6–6.6 × 10^9^/L; Lymph = 0.5–3.8 × 10^9^/L; Mono = < 1.2 × 10^9^/L; ALP = 30–564 U/L; ALT = 5–21 U/L; ALB = 34–50 g/L; CRE = 80–150 μmol/L; Glob = 18–30 g/L; TP = 58–73 g/L; GGT = 6–17 U/L; BUN = 1–8 mmol/L. Reference ranges were retrieved from Canfield et al. [104] and Vetnostics. Abbreviations: WBC = white blood cells; RBC = red blood cells; HCT = haematocrit test; Neut = neutrophils; Lymph = lymphocytes; Mono = monocytes; ALP = alkaline phosphatase; ALT = alanine transaminase; ALB = albumin; CRE = creatinine; Glob = globulins; TP = total protein; GGT = gamma-glutamyl transferase; BUN = blood urea nitrogen. Information is not available for blank cells. Values outside the reference range are in red.
(PDF)

S5 Table Pairwise comparison of the biochemical analytes between clinically normal, diseased, and ‘other’ koalas. ^1^ = reference category; SD = standard deviation. Statistically significant value is bolded.
(PDF)

S6 Table Univariable results showing the KYN, TRP, and KYN:TRP ratio between clinically normal (n = 35) versus diseased (n = 35), clinically normal versus ‘other’ (n = 10), and diseased versus ‘other’ groups. ^1^ = reference category; ^α^ = non-normal distribution; ^β^ = normal distribution; SD = standard deviation. Statistically significant value is bolded.
(PDF)

S1 Fig Scatterplot of KYN concentrations (reference range: 0.34–1.23 μg/mL), TRP concentrations (reference range: 4.27–10.4 μg/mL), and KYN:TRP ratios (reference range: 0.05–0.22) in clinically normal, diseased, and ‘other’ koalas. (A) KYN concentrations. (B) TRP concentrations. (C) KYN:TRP ratios. Each point represents an individual koala. The green lines define the reference range interval. The outlier for KYN and the KYN:TRP ratio in the ‘other’ group is the same koala with leukaemia. The outlier for the KYN:TRP ratio in the ‘diseased’ group had both eyes and UGT clinical signs with mammary mass.
(TIF)

S2 Fig ROC curves of the KYN, TRP and KYN:TRP ratio in clinically normal, diseased, and ‘other’ koalas. Abbreviations: KYN = kynurenine; TRP = tryptophan; concen = concentration.
(TIF)

S3 Fig ROC curves of the KYN, TRP and KYN:TRP ratio in the released and euthanised koalas of the diseased and ‘other’ groups. Abbreviations: KYN = kynurenine; TRP = tryptophan; concen = concentration.
(TIF)
