# Supplementary material for: Preliminary analyses of tryptophan, kynurenine, and the kynurenine: Tryptophan ratio in plasma, as potential biomarkers for systemic chlamydial infections in koalas
Source: PLoS One. 2024 Dec 19;19(12):e0314945. doi: 10.1371/journal.pone.0314945 (PMC11658483; doi:10.1371/journal.pone.0314945)
Supplement: S1 Table — (PDF) [file pone.0314945.s001.pdf]

Intra-day 1	KYN			TRP		
Expected concentration (μg/mL)	2.50	5.00	20.0	2.50	5.00	20.0
Estimated concentration (μg/mL)	2.39 ± 0.05	4.56 ± 0.30	20.7 ± 2.30	2.66 ± 0.18	4.81 ± 0.23	21.1 ± 0.51
Accuracy (%)	93.7 - 97.6	84.7 – 96.5	92.6 – 116	98.3 – 113	92.2 – 101	103 – 108
Precision (%)	2.05	6.54	11.1	6.95	4.71	2.41
**Intra-day 2**						
Expected concentration (μg/mL)	2.50	5.00	20.0	2.50	5.00	20.0
Estimated concentration (μg/mL)	2.81 ± 0.11	4.93 ± 0.20	20.0 ± 1.37	2.53 ± 0.20	4.99 ± 0.48	20.0 ± 1.82
Accuracy (%)	110 – 118	95.0 – 103	92.3 – 105	91.9 – 107	89.5 – 109	89.8 – 107
Precision (%)	3.92	3.98	6.85	8.06	9.70	9.11
**Intra-day 3**						
Expected concentration (μg/mL)	2.50	5.00	20.0	2.50	5.00	20.0
Estimated concentration (μg/mL)	2.71 ± 0.22	4.75 ± 0.50	20.0 ± 1.82	2.27 ± 0.19	5.26 ± 0.60	20.0 ± 2.76
Accuracy (%)	103 – 119	84.3 – 104	94.0 – 111	85.2 – 99.4	91.8 – 115	89.0 – 115
Precision (%)	8.06	10.5	9.09	8.16	11.5	13.8
**Inter-day**						
Expected concentration (μg/mL)	2.50	5.00	20.0	2.50	5.00	20.0
Estimated concentration (μg/mL)	2.64 ± 0.13	4.75 ± 0.33	20.2 ± 1.83	2.49 ± 0.19	5.02 ± 0.44	20.4 ± 1.70
Accuracy (%)	103 – 108	91.7 – 97.2	93.6 – 110	95.1 – 103	94.2 – 105	96.1 – 110
Precision (%)	4.68	7.00	9.03	7.73	8.63	8.45
