# Supplementary material for: Preliminary analyses of tryptophan, kynurenine, and the kynurenine: Tryptophan ratio in plasma, as potential biomarkers for systemic chlamydial infections in koalas
Source: PLoS One. 2024 Dec 19;19(12):e0314945. doi: 10.1371/journal.pone.0314945 (PMC11658483; doi:10.1371/journal.pone.0314945)
Supplement: S2 Table — (PDF) [file pone.0314945.s002.pdf]

Koala	Sex	Maturity	Age (yrs)	Location	Clinically normal (Y/N)	Site/s of infection or other co-morbidities	Outcome	KYN concentration (µg/mL)	TRP concentration (µg/mL)	KYN:TRP ratio
K1	F	Adult	7	NSW	Y		Released	1.20	9.22	0.13
K2	F	Adult	2	NSW	N	Eyes	Released	1.32	10.17	0.13
K3	M	Adult	4	NSW	Y		Released	0.87	6.30	0.14
K4	F	Juvenile	1	NSW	Y		Released	2.00	10.75	0.19
K5	M	Adult	6	NSW	Y		Released	0.86	8.65	0.10
K6	M	Adult	7	NSW	Y		Released	0.65	8.90	0.07
K7	F	Adult	10	NSW	Y		Released	0.74	7.30	0.10
K8	M	Juvenile	1	NSW	Y		Released	1.11	9.58	0.12
K9	F	Adult	14	NSW	Y		Released	0.77	9.93	0.08
K10	F	Adult	8	NSW	Y		Released	0.62	9.50	0.07
K11	M	Adult	9	NSW	Y		Released	0.67	7.34	0.09
K12	M	Adult	4	NSW	Y		Released	0.65	5.97	0.11

K13	M	Juvenile	1	NSW	Y		Released	0.76	10.08	0.08
K14	M	Adult	5	NSW	Y		Released	0.65	7.09	0.09
K15	F	Adult	15	NSW	Y		Released	0.67	7.26	0.09
K16	F	Adult	6	NSW	Y		Released	1.10	6.37	0.17
K17	F	Adult	4	NSW	N	UGT	Released	0.86	6.83	0.13
K18	M	Juvenile	1	NSW	Y		Released	0.48	6.11	0.08
K19	M	Adult	7	NSW	N	Other	Released	1.11	9.54	0.12
K20	M	Adult	4	NSW	N	Liver disease, retrovirus	Released	0.93	8.81	0.11
K21	F	Adult	14	NSW	Y		Released	0.75	8.72	0.09
K22	M	Adult	10	NSW	Y		Released	0.77	6.79	0.11
K23	F	Adult	16	NSW	Y		Released	0.54	7.69	0.07
K24	M	Juvenile	1.5	NSW	Y		Released	0.78	7.40	0.11
K25	M	Adult	5	NSW	N	Joints infection	Admitted	0.71	6.94	0.10
K26	M	Adult	4	NSW	N	Trauma	Released	1.58	8.15	0.19
K27	M	Juvenile	2	NSW	N	Eyes	Released	0.88	7.85	0.11

K28	M	Adult	12	NSW	Y		Released	0.95	6.22	0.15
K29	M	Adult	14	NSW	Y		Released	0.50	6.77	0.07
K30	M	Adult	3	NSW	Y		Released	0.40	5.96	0.07
K31	M	Adult		NSW	N	Eyes	Admitted	0.79	5.71	0.14
K32	F	Adult	3	NSW	Y		Released	0.63	7.02	0.09
K33	M	Juvenile	2	NSW	Y		Released	0.76	7.22	0.11
K34	M	Adult	3.5	NSW	Y		Admitted	0.43	4.78	0.09
K35	F	Adult	8	NSW	Y		Admitted	0.54	6.26	0.09
K36	M	Adult	2.5	NSW	Y		Admitted	0.87	5.60	0.16
K37	F	Adult	3	NSW	Y		Released	0.87	4.68	0.19
K38	F	Adult	12+	NSW	Y		Released	1.08	5.82	0.19
K39	F	Adult	9	NSW	N	Eyes, UGT, mammary mass	Euthanised	1.96	3.17	0.62
K40	M	Adult	4	NSW	N	Eyes, UGT	Admitted	1.13	4.39	0.26
K41	F	Adult	12+	NSW	N	UGT	Released	1.51	9.05	0.17
K42	M	Adult	12+	NSW	N	Other	Euthanised	2.29	6.65	0.34
K43	F	Adult	6	NSW	N	UGT	Euthanised	0.59	3.55	0.17

K44	F	Adult	9	NSW	N	Eyes, UGT	Euthanised	1.18	4.18	0.29
K45	F	Adult	7	NSW	Y		Released	1.32	5.65	0.23
K46	M	Adult	8	NSW	N	Eyes, UGT	Euthanised	1.78	7.19	0.25
K47	M	Adult	5	NSW	N	Eyes, UGT	Euthanised	1.22	7.67	0.16
K48	M	Adult	3	NSW	N	Eyes	Euthanised	1.30	4.21	0.31
K49	F	Adult	6	NSW	N	UGT	Euthanised	1.44	5.79	0.25
K50	M	Adult	7	NSW	N	Eyes	Euthanised	1.37	6.55	0.21
K51	F	Adult	3	NSW	Y		Released	0.68	7.58	0.09
K52	F	Adult	10+	NSW	N	UGT	Euthanised	1.75	9.18	0.19
K53	F	Adult	2.5	NSW	Y		Released	0.89	7.71	0.12
K54	M	Adult	6	NSW	N	Advanced alopecia, skin infection	Euthanised	1.41	6.60	0.21
K55	F	Adult	4	NSW	N	Eyes, UGT	Euthanised	1.19	6.60	0.18
K56	F	Adult	9	NSW	N	Eyes, UGT	Euthanised	1.32	6.70	0.20
K57	F	Adult	12+	NSW	N	Eyes, UGT	Admitted	1.08	6.10	0.18
K58	M	Adult	10+	NSW	N	Other	Euthanised	3.79	4.43	0.85

K59	F	Adult	5	NSW	N	Trauma	Released	0.99	3.81	0.26
K60	M	Adult	8	NSW	N	Eyes	Admitted	1.13	7.39	0.15
K61	F	Adult	5	NSW	N	Eyes, UGT	Euthanised	1.98	6.38	0.31
K62	F	Adult	8	NSW	N	Leukaemia	Released	6.38	4.72	1.35
K63	M	Adult	8	NSW	N	Eyes	Admitted	0.87	7.76	0.11
K64	F	Adult	8	NSW	N	UGT	Euthanised	0.68	4.28	0.16
K65	M	Adult	6	QLD	N	UGT	Euthanised	1.95	7.10	0.27
K66	F	Juvenile	1.5	QLD	N	Eyes	Released	2.01	7.03	0.29
K67	F	Adult	4	QLD	N	UGT	Released	1.65	6.38	0.26
K68	F	Adult	7	QLD	N	Eyes	Euthanised	1.71	3.60	0.48
K69	F	Adult	4	QLD	N	UGT	Admitted	0.82	8.22	0.10
K70	M	Adult	3	QLD	Y		Released	0.82	8.73	0.09
K71	M	Adult	4	QLD	N	Eyes, UGT	Released	0.88	6.88	0.13
K72	F	Adult	6	QLD	N	Eyes, UGT	Released	0.94	5.69	0.17
K73	M	Adult	4	QLD	N	UGT	Released	0.88	3.64	0.24
K74	F	Adult	8	QLD	N	Abdominal lymphoma	Euthanised	1.32	4.75	0.28
K75	F	Adult	3	QLD	N	UGT	Released	0.51	3.55	0.14

K76	M	Adult	8	QLD	Y		Released	1.07	5.59	0.19
K77	F	Adult	4	QLD	N	UGT	Released	0.82	2.20	0.37
K78	F	Adult	5	QLD	N	Eyes, UGT	Released	0.94	3.99	0.24
K79	F	Adult	2	QLD	N	UGT	Released	0.95	5.17	0.18
K80	M	Adult	5	QLD	N	UGT	Released	0.50	3.49	0.14
