# Supplementary material for: Preliminary analyses of tryptophan, kynurenine, and the kynurenine: Tryptophan ratio in plasma, as potential biomarkers for systemic chlamydial infections in koalas
Source: PLoS One. 2024 Dec 19;19(12):e0314945. doi: 10.1371/journal.pone.0314945 (PMC11658483; doi:10.1371/journal.pone.0314945)
Supplement: S3 Table — Non-normally distributed data was analysed using non-parametric test. 1 = reference category; α = non-normal distribution; β = normal distribution; SD = standard deviation. Statistically significant value is bolded. (PDF) [file pone.0314945.s003.pdf]

| **Biomarker** | **Maturity** | **Mean ± SD** | ***P* value** |
|---|---|---|---|
| KYN concentration (μg/mL) [α] | Juvenile [1] | 0.98 ± 0.54 | 0.45 |
| | Adult | 0.78 ± 0.22 | |
| TRP concentration (μg/mL) [β] | Juvenile [1] | 8.53 ± 1.86 | **0.04** |
| | Adult | 7.08 ± 1.40 | |
| KYN:TRP ratio [α] | Juvenile [1] | 0.11 ± 0.04 | 0.88 |
| | Adult | 0.12 ± 0.04 | |
