# Supplementary material for: Preliminary analyses of tryptophan, kynurenine, and the kynurenine: Tryptophan ratio in plasma, as potential biomarkers for systemic chlamydial infections in koalas
Source: PLoS One. 2024 Dec 19;19(12):e0314945. doi: 10.1371/journal.pone.0314945 (PMC11658483; doi:10.1371/journal.pone.0314945)
Supplement: S4 Table — Reference ranges: WBC = 2.6–9.8 × 10^9^/L; RBC = 2.9–4.4 × 10^12^/L; HCT = 0.31–0.45 L/L; Neut = 0.6–6.6 × 10^9^/L; Lymph = 0.5–3.8 × 10^9^/L; Mono = < 1.2 × 10^9^/L; ALP = 30–564 U/L; ALT = 5–21 U/L; ALB = 34–50 g/L; CRE = 80–150 μmol/L; Glob = 18–30 g/L; TP = 58–73 g/L; GGT = 6–17 U/L; BUN = 1–8 mmol/L. Reference ranges were retrieved from Canfield et al. [104] and Vetnostics. Abbreviations: WBC = white blood cells; RBC = red blood cells; HCT = haematocrit test; Neut = neutrophils; Lymph = lymphocytes; Mono = monocytes; ALP = alkaline phosphatase; ALT = alanine transaminase; ALB = albumin; CRE = creatinine; Glob = globulins; TP = total protein; GGT = gamma-glutamyl transferase; BUN = blood urea nitrogen. Information is not available for blank cells. Values outside the reference range are in red. (PDF) [file pone.0314945.s004.pdf]

	Haematology							Biochemistry							
**Koala**	**Health status**	**WBC (10^9/L)**	**RBC (10^12/L)**	**HCT (L/L)**	**Neut (10^9/L)**	**Lymph (10^9/L)**	**Mono (10^9/L)**	**ALP (U/L)**	**ALT (U/L)**	**ALB (g/L)**	**CRE (µmol/L)**	**Glob (g/L)**	**TP (g/L)**	**GGT (U/L)**	**BUN (mmol/L)**
K2	Diseased	8.51	2.50	0.27	4.19	3.18	0.24	185.4	16.6	36.6	48.6	23.8	60.4	8.96	1.96
K10	Normal	6.30	2.68	0.31	4.57	1.25	0.24	74.5	12.3	37.4	85.6	25.7	63.1	15.8	2.69
K12	Normal	6.35	2.83	0.33	2.03	3.87	0.29	127	22.3	36.5	54.2	19.8	56.2	16.9	2.15
K13	Normal	10.4	2.69	0.32	8.82	1.22	0.19	228	16.5	37.3	70.1	18.5	55.7	6.83	2.72
K14	Normal	16.0	3.46	0.39	8.88	5.50	0.69	165.8	1.81	40.4	90.1	23.6	64.0	12.4	2.43
K15	Normal	5.01	2.06	0.25	4.24	0.00	0.39	83.8	3.69	37.6	51.0	27.3	64.9	9.51	1.48
K16	Normal	10.3	2.14	0.27	6.20	2.27	0.57	161	10.4	33.8	60.7	26.4	60.2	11.9	2.43
K17	Diseased	5.86	2.91	0.31	2.16	2.89	0.50	96.0	4.23	36.6	58.7	28.1	64.7	6.92	1.35
K37	Normal	13.8	2.90	0.32	9.40	4.10	0.30	175	17.0	41.0	100	21.0	62.0	13.0	1.68
K38	Normal	5.40	2.80	0.29	3.50	1.10	0.10	35.0	11.0	42.0	96.0	28.0	70.0	10.0	2.29
K39	Diseased	4.10	1.80	0.17	3.60	0.40	0.10	41.0	37.0	30.0	62.0	30.0	60.0	9.00	0.47
K40	Diseased	5.30	3.00	0.37	1.40	3.20	0.30	100	< 5.00	40.0	82.0	26.0	66.0	7.00	0.28
K41	Diseased	6.70	3.10	0.32	3.00	2.70	0.10	67.0	10.0	41.0	122	32.0	73.0	12.0	0.28
K42	Other	8.00	3.00	0.33	7.40	0.30	0.20	67.0	17.0	35.0	88.0	31.0	66.0	10.0	2.15
K43	Diseased	5.60	2.50	0.26	2.70	2.60	0.20	20.0	11.0	25.0	57.0	17.0	42.0	< 5.00	0.98
K44	Diseased	5.60	3.40	0.33	3.00	2.20	0.10	96.0	8.0	33.0	93.0	28.0	61.0	10.0	0.14

K45	Normal	3.60	3.20	0.34	0.80	2.40	0.10	67.0	15.0	39.0	117	27.0	66.0	11.0	0.56
K46	Diseased	5.80	3.40	0.31	2.00	2.80	0.40	43.0	13.0	41.0	112	25.0	66.0	14.0	1.17
K47	Diseased	4.50	3.50	0.36	3.70	0.60	0.00	185	59.0	41.0	106	27.0	68.0	11.0	2.48
K48	Diseased							194	34.0	39.0	81.0	26.0	65.0	12.0	4.25
K49	Diseased	10.1	2.90	0.35	5.00	3.10	0.40	61.0	14.0	40.0	102	27.0	67.0	15.0	0.56
K50	Diseased	2.50	4.20	0.40	1.80	0.70	0.10	127	31.0	46.0	114	28.0	74.0	7.00	6.22
K51	Normal	1.20	2.40	0.23	0.60	0.60	0.00	64.0	9.00	33.0	108	19.0	52.0	8.00	0.33
K52	Diseased	3.50	3.40	0.32	1.30	1.10	0.10	109	6.0	39.0	116	29.0	68.0	11.0	1.12
K53	Normal	5.80	3.10	0.33	0.90	3.90	0.10	242	13.0	39.0	104	20.0	59.0	10.0	1.96
K54	Other	13.8	2.70	0.26	11.7	1.90	0.10	38.0	9.00	35.0	69.0	43.0	78.0	5.00	5.56
K55	Diseased	3.00	3.00	0.32	0.50	0.60	0.00	128	7.00	37.0	89.0	29.0	66.0	14.0	0.56
K56	Diseased	23.9	3.70	0.40	19.8	2.90	1.20	274.0	16.0	47.0	64.0	31.0	78.0	13.0	4.58
K57	Diseased	5.30	3.30	0.34	2.80	1.90	0.30	94.0	14.0	42.0	102	31.0	73.0	14.0	0.61
K58	Other	6.30	3.60	0.37	4.30	1.80	0.10	32.0	5.00	42.0	84.0	28.0	70.0	7.00	4.44
K59	Other	2.60	4.40	0.42	1.50	1.00	0.10	60.0	48.0	37.0	119	22.0	59.0	< 5.00	0.23
K60	Diseased	3.90	3.50	0.36	2.50	1.10	0.20	118.0	18.0	43.0	85.0	34.0	77.0	8.00	1.36
K61	Diseased	8.30	3.40	0.39	5.00	2.80	0.30	86.0	53.0	42.0	65.0	29.0	71.0	12.0	1.17
K62	Other	649	0.90	0.10	0.00	13.0	0.00	51.0	115	28.0	70.0	25.0	53.0	14.0	4.39
K63	Diseased	6.60	3.60	0.38	3.20	3.10	0.20	166	10.0	39.0	93.0	36.0	75.0	14.0	0.42
K64	Diseased	8.30	3.00	0.30	5.30	2.90	0.10	19.0	44.0	28.0	86.0	23.0	51.0	< 5.00	1.40
