# Supplementary material for: Preliminary analyses of tryptophan, kynurenine, and the kynurenine: Tryptophan ratio in plasma, as potential biomarkers for systemic chlamydial infections in koalas
Source: PLoS One. 2024 Dec 19;19(12):e0314945. doi: 10.1371/journal.pone.0314945 (PMC11658483; doi:10.1371/journal.pone.0314945)
Supplement: S5 Table — 1 = reference category; SD = standard deviation. Statistically significant value is bolded. (PDF) [file pone.0314945.s005.pdf]

| **Parameter** | **Experimental groups** | **Mean ± SD** | ***P* value** |
|---|---|---|---|
| Glob (g/L) | Clinically normal [1] | 23.3 ± 3.71 | **0.01** |
| | Diseased | 28.0 ± 4.13 | |
| | 'Other' | 29.8 ± 8.11 | 0.17 |
| | Diseased vs. 'other' | | 1.00 |
| ALP (U/L) | Clinically normal [1] | 129 ± 70.0 | 0.86 |
| | Diseased | 111 ± 65.0 | |
| | 'Other' | 49.6 ± 14.6 | **0.03** |
| | Diseased vs. 'other' | | 0.07 |
