# Supplementary material for: Preliminary analyses of tryptophan, kynurenine, and the kynurenine: Tryptophan ratio in plasma, as potential biomarkers for systemic chlamydial infections in koalas
Source: PLoS One. 2024 Dec 19;19(12):e0314945. doi: 10.1371/journal.pone.0314945 (PMC11658483; doi:10.1371/journal.pone.0314945)
Supplement: S6 Table — 1 = reference category; α = non-normal distribution; β = normal distribution; SD = standard deviation. Statistically significant value is bolded. (PDF) [file pone.0314945.s006.pdf]

**Biomarker**	**Health status**	**Mean ± SD**	***P* value**
KYN concentration (μg/mL) [α]	Clinically normal [1]	0.81 ± 0.30	**< 0.001**
	Diseased	1.20 ± 0.44	
	'Other'	2.05 ± 1.77	**< 0.001**
	Diseased vs. 'Other'		0.16
TRP concentration (μg/mL) [β]	Clinically normal [1]	7.33 ± 1.56	**0.001**
	Diseased	5.93 ± 1.94	
	'Other'	6.44 ± 1.98	0.14
	Diseased vs. 'Other'		0.47
KYN:TRP ratio [α]	Clinically normal [1]	0.11 ± 0.04	**< 0.001**
	Diseased	0.22 ± 0.11	
	'Other'	0.38 ± 0.41	**< 0.001**
	Diseased vs. 'Other'		0.49
