# Supplementary figures and images for: Preliminary analyses of tryptophan, kynurenine, and the kynurenine: Tryptophan ratio in plasma, as potential biomarkers for systemic chlamydial infections in koalas
Source: PLoS One. 2024 Dec 19;19(12):e0314945. doi: 10.1371/journal.pone.0314945 (PMC11658483; doi:10.1371/journal.pone.0314945)

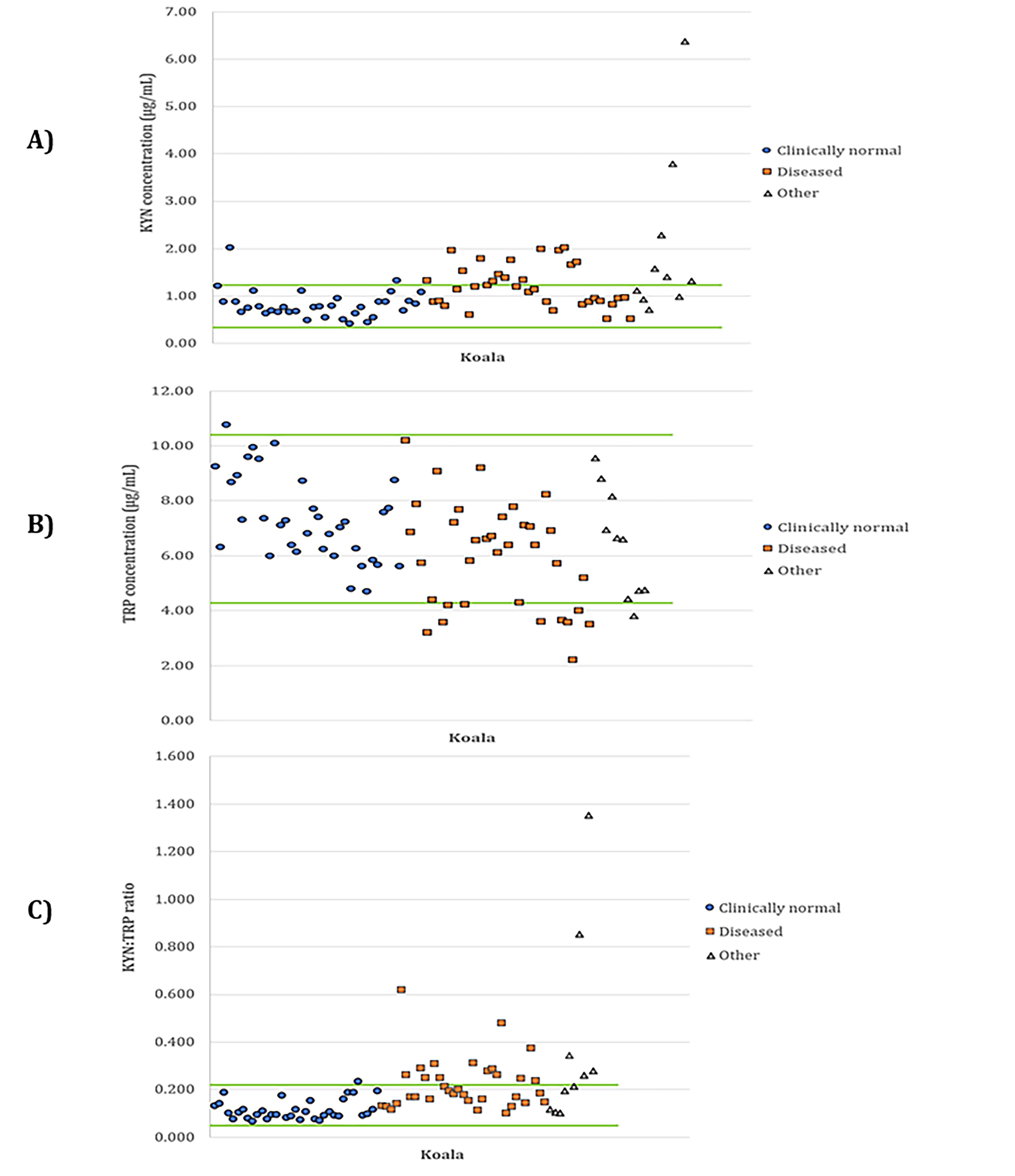

Supplement: S1 Fig — (A) KYN concentrations. (B) TRP concentrations. (C) KYN:TRP ratios. Each point represents an individual koala. The green lines define the reference range interval. The outlier for KYN and the KYN:TRP ratio in the ‘other’ group is the same koala with leukaemia. The outlier for the KYN:TRP ratio in the ‘diseased’ group had both eyes and UGT clinical signs with mammary mass. (TIF) [file pone.0314945.s007.tif]

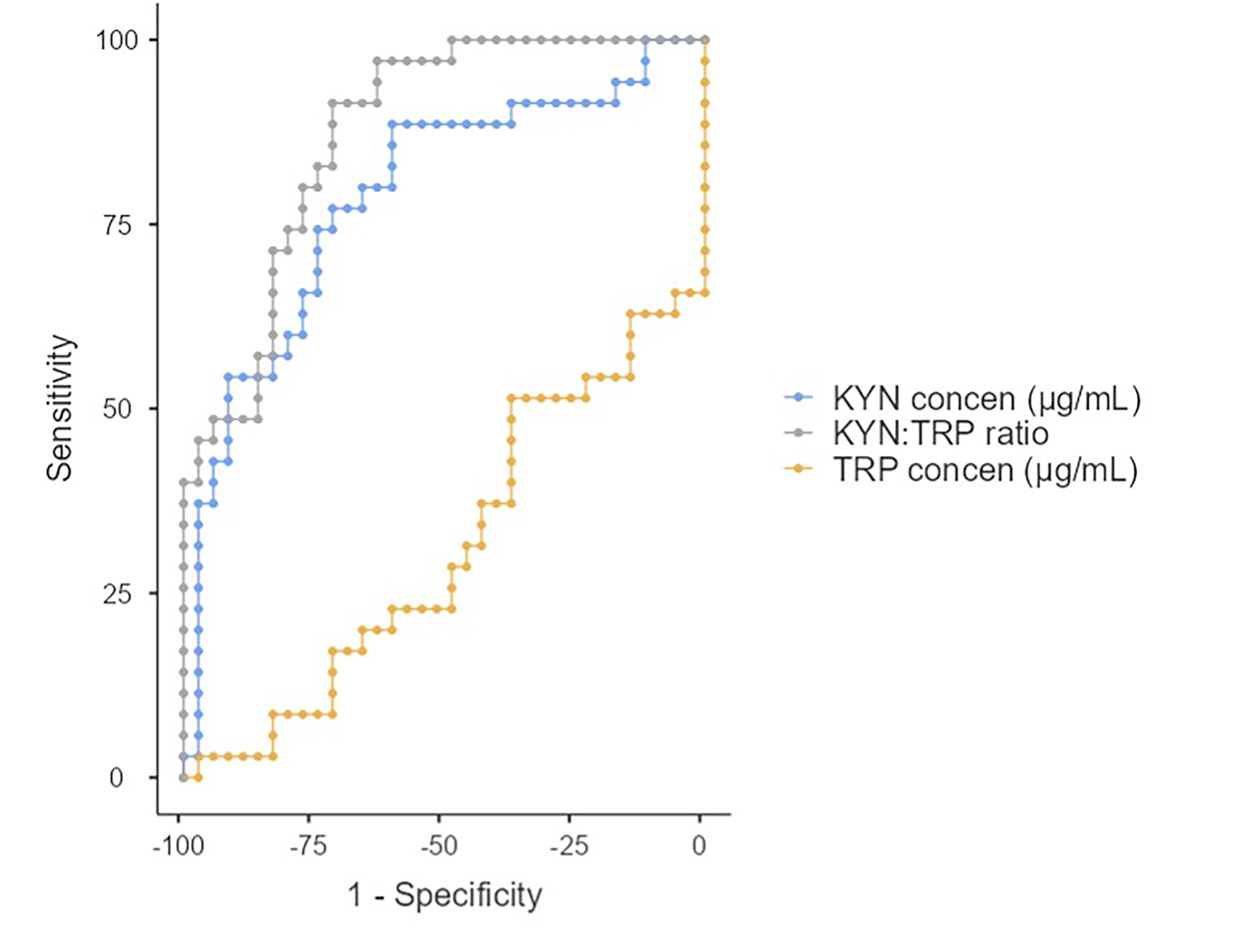

Supplement: S2 Fig — Abbreviations: KYN = kynurenine; TRP = tryptophan; concen = concentration. (TIF) [file pone.0314945.s008.tif]

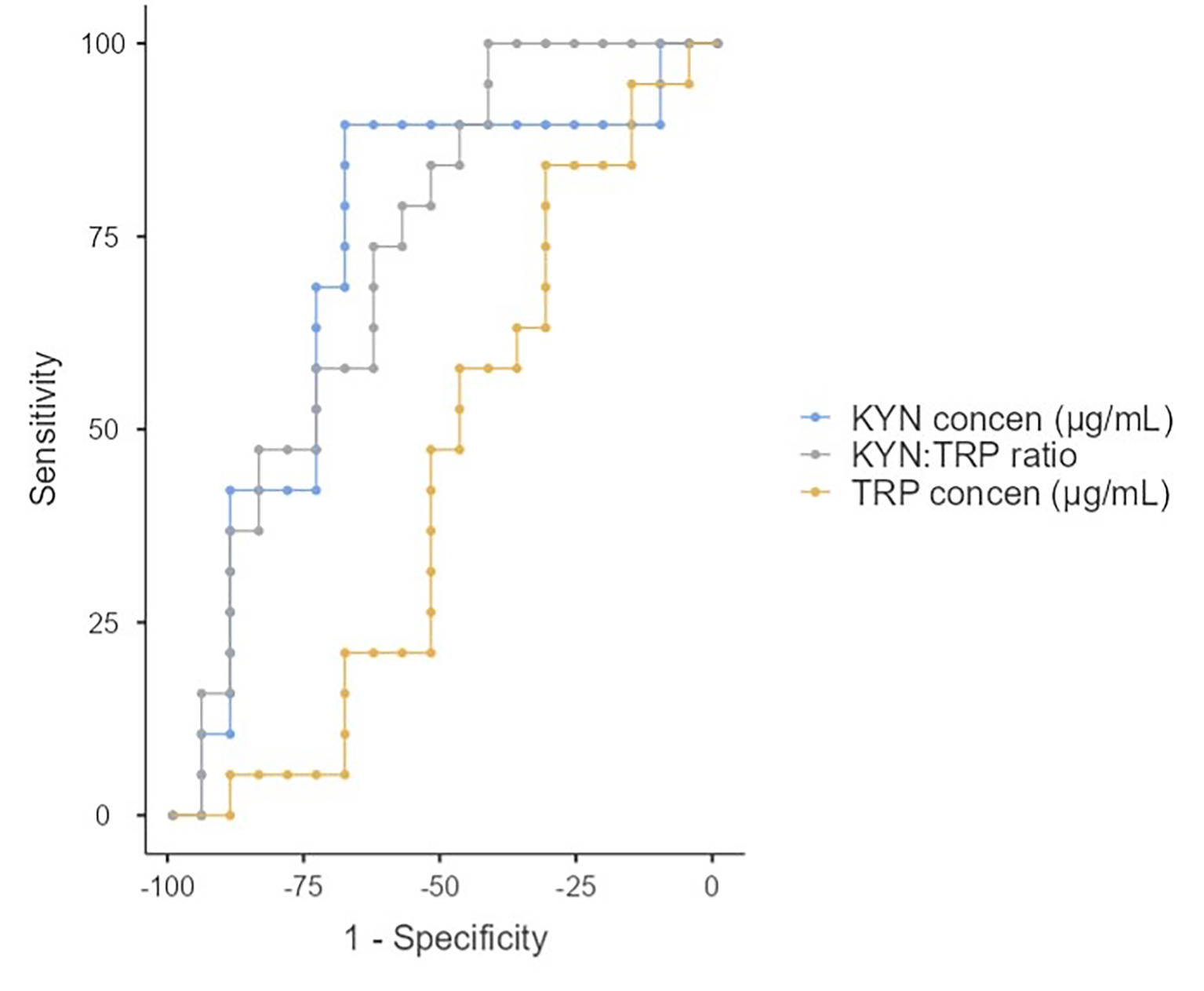

Supplement: S3 Fig — Abbreviations: KYN = kynurenine; TRP = tryptophan; concen = concentration. (TIF) [file pone.0314945.s009.tif]
